# Randomized, Placebo-Controlled Clinical Trial of Omega-3 as Supplemental Treatment in Schizophrenia

**DOI:** 10.5539/gjhs.v6n7p103

**Published:** 2014-09-18

**Authors:** Hamidreza Jamilian, Hasan Solhi, Mehri Jamilian

**Affiliations:** 1 Department of Psychiatry, Arak University of Medical Sciences, Arak, Iran; 2 Department of Obstetrics and Gynecology, Arak University of Medical Sciences, Arak, Iran

**Keywords:** Schizophrenia, Omega-3, PANSS, supplement therapy

## Abstract

**Introduction:**

Recent studies found omega-3 fatty acid deficiency in brain cell membranes of schizophrenic patients. Conventional antipsychotics have many adverse reactions. Safety, availability and low price made omega-3 as a potential supplement for treatment of these patients. This study investigated the efficacy of omega-3 fatty acid as add-on treatment in schizophrenia.

**Material & Methods:**

A randomized, double blind, placebo controlled fixed-dose, add-on clinical trial conducted over 8 weeks. 60 patients with documented schizophrenia randomly divided into two groups: omega-3(1000 mg/day) (n=30) and placebo (n=30). Patients received omega-3 or placebo in addition to their standard antipsychotic treatment. Patient follow up was done using Positive and Negative syndrome Scale (PANSS). Data analyzed using SPSS software v.20.

**Result:**

At the end of 8 weeks treatment, PANSS score decreased significantly in both groups (p<0.05) in comparison to baseline. Efficacy of omega-3 in decreasing general psychopathologic and total scores was significant in comparison to placebo group from 4 and 6 weeks after onset of treatment, respectively (p<0.05). Totally, omega-3 supplement therapy efficacy in comparison to sole conventional antipsychotics was 0.86 which was not significant (p>0.05).

**Conclusion:**

We found that supplemental omega-3 might increase efficacy of conventional antipsychotics in decreasing symptoms of schizophrenia. Low price, rare adverse reactions and availability of omega-3 made this substance a potential supplement in improved treatment of schizophrenia.

## 1. Introduction

The etiology of schizophrenia and psychotic symptoms are largely unknown. Genetic factors are important (Lichtenstein et al., 2009), but environmental factors such as unhealthy lifestyle with a poor diet may be responsible (Samele et al., 2007). Recent studies found some evidences that dietary level of essential fatty acids (EFAs), may affect the occurrence and course of schizophrenia (Peet et al., 2001; Mellor, Laugharne, & Peet, 1995). There are two types of EFAs, omega-6 and omega-3. Omega-3 is abundant in oily fish such as mackerel and sardines.

A recent meta-analysis study found that prevalence of schizophrenia was greater for groups with low fish consumption (Kinney et al., 2009).

Besides, it has previously shown that low consumption of fish and seafood during pregnancy may increase the risk for a low IQ and impaired neuro-developmental outcomes in childhood (Hibbeln et al., 2007), which in turn may associated with an increased risk for mental disorders like schizophrenia in adulthood (Koenen et al., 2009). On the other hand, several studies have shown that schizophrenic patients often have low levels of the particular EFAs, which seems to be necessary for normal nerve cell-membrane metabolism. The evidence is strong enough to have led to a ‘membrane phospholipid hypothesis’ of schizophrenia (Glen et al., 1994; Horrobin, 1998a; Horrobin, 1998b)

Although the mainstay of schizophrenia treatment is pharmacological targeting neurotransmission within the brain, extrapyramidal symptoms and limited efficacy of conventional antipsychotic drugs are serious limitations for consumption of these drugs. On the other hand, high acquisition costs of new generations of antipsychotics have put these drugs beyond the reach of patients in lower-income countries (Emsley et al., 1999).

So, given the basis of a potential correlation between schizophrenia and low fatty acid levels in the brain of schizophrenic patients, it seems reasonable and worthwhile to test the idea that dietary supplementation of omega-3 may be beneficial in treatment of these patients. In addition, omega-3 supplementation may enhance the efficacy of commonly used antipsychotic drugs already due to altered neurotransmission.

The objective of the present study was to investigate the efficacy of oral supplement of omega-3 in treatment of patients with schizophrenia.

## 2. Material & Methods

This clinical trial submitted to Iranian Registry of Clinical Trials (IRCT) No. IRCT201202117373N2 on 27.02.2012. In our Triple-blind, placebo-controlled clinical trial, 60 schizophrenic patients were enrolled. Schizophrenia diagnosis done after clinical interview with each patient by a psychiatrist based on DSM-IV diagnosis criteria. The aim of study explained for the patients and their legal guardians, and then they were requested to fill out the informed consent form for participation in our clinical trial. Patients randomly and equally divided into two equal groups: Control (n=30) and Omega-3 (n=30). Patients of both groups treated with one of standard atypical antipsychotic drugs (olanzapine, risperidone and clozapine). In addition to standard antipsychotic drug, patients of omega-3 group received 1000 mg per day of omega 3 while patients in control group received placebo at the same time in the capsules similar to omega 3 capsules.

Patients of both groups received treatment for a period of 8 weeks. Patient follow up was done using Positive and Negative Syndrome Scale (PANSS). A nurse from psychiatry ward trained for this study for prescription of the drugs and a psychiatrist evaluated of PANSS score during the follow up period.

Score of each patient registered in prepared forms before the onset of treatment and after 2, 4, 6 and 8 weeks of drug consumption. The PANSS score is a currents and well-known hand scored instrument used for measuring symptom severity of patients with schizophrenia (Kay, Opler, & Fiszbein, 1987). It is widely used in the study of antipsychotic therapy.

To assess a patient using PANSS, an approximately 45-minute clinical interview is conducted. The patient is rated from one to seven on 30 different symptoms based on the interview as well as reports of family members or primary care hospital workers. The scale consists of three general interview items (7 items for Positive scale, 7 items for Negative scale and 16 items for General Psychopathology scale) (Kay, 1991). The main outcome of our study was evaluation of changes in PANSS scale items during treatment in comparison to values before the beginning of study.

Patients’ inclusion criteria for our clinical trial consist of: 1. filling out informed consent, 2. Ages between 15-55 year old, 3. Diagnosis of schizophrenia based on DSM-IV-TR criteria by a psychiatrist and 4. Total PANSS score of at least 60. The exclusion criteria were: Pregnancy or lactating period, drug abuse, severe physical disease and contraindication of omega-3 consumption. Data were analyzed using SPSS software v.20. Data were considered significant at the level of P<0.05 (Dalfard & Ranjbar, 2012).

## 3. Result

In our double-blind clinical trial, 60 patients were involved with definite diagnosis of schizophrenia. The mean age of patients were 31.51 (SD=7.9) y/o. thirty-one (51.7%) of patients were male and 29 (48.3%) were female. There was no significant difference between two groups among their age and sex. Duration of illness in control group was 10.11 (SD=5.24) year in comparison to 9.30 (SD=5.03) year of case group. The mean Admission number in placebo and omega-3 groups were 4.43 (SD=1.90) and 3.93 (SD=1.61), respectively (P>0.05) (Table 1).

**Table 1:** Characteristics of the patients

	**Placebo (n=30)**	**Omega-3 (n=30)**
**Age (y/o)**	31.01±8.81	32.01±7.13
**Sex**		
**Male**	15 (50%)	16 (53.3%)
**Female**	15 (50%)	14 (46.7%)
**Duration of illness (year)**	10.11±5.24	9.30±5.03
**Admission number**	4.43±1.90	3.93±1.61

The mean of positive, negative, general and total scores of patients of each group during the weeks 0,2,4,6 and 8 after onset of treatment are shown in Table 2. Baseline PANSS scores (week 0) had no significant difference between groups except general scores. Among both groups, Positive, negative and total score were decreased from week 0 to 8. This decrement is significant from week 2 to 8 for positive, negative and total score in comparison to week 0 (p<0.0001).

**Table 2:** Effect of 8 weeks treatment with omega-3 supplement or placebo on PANSS score

	Positive score (mean±SD)	Negative score (mean±SD)	General score (mean±SD)	Total score (mean±SD)
**Week 0**				
**Placebo**	27.63±3.94	23.06±3.64	48.16±3.90	98.26±4.51
**Omega-3**	26.66±3.33	23.83±3.35	45.90±4.68	96.13±9.61
**Week 2**				
**Placebo**	24.96±3.30	21.56±3.16	37.43±4.24	83.96±4.36
**Omega-3**	23.96±3.58	21.73±2.57	35.56±4.50	81.20±7.97
**Week 4**				
**Placebo**	21.56±3.59	14.96±2.41*	33.20±4.59*	70.06±5.57
**Omega-3**	20.96±3.28	16.40±2.78*	30.06±3.59*	66.70±7.99
**Week 6**				
**Control**	17.50±3.45	12.96±2.93	29.73±2.85*	60.20±3.41*
**Case**	16.16±2.93	14.03±2.77	26.06±3.34*	56.23±5.76*
**Week 8**				
**Placebo**	14.66±2.48	11.26±2.80	26.70±3.46*	52.43±3.32*
**Omega-3**	14.00±2.79	12.13±2.59	22.33±3.46*	49.13±5.31*

**Table 3:** 

w_neg	Coef.	Std. Err.	z	P Value	[95% Interval]	Conf.
****Group 1 compared to 0****	0.86	0.717	1.2	0.231	-0.546	2.27

Based on GEE population-averaged model, omega-3 effect on decreasing the PANSS score was 0.86 of standard treatment for schizophrenia (SE=0.717, Z=1.2, 95% Conf. Interval=-0.546), but this difference was non-significant (p=0.231).

There was no significant different between two groups among sex, age, duration of illness and admission number. All data demonstrated as mean±SD except sex.Data considered significant at the level of p<0.05.

All scores decreased after 8 weeks treatment in comparison to week 0 (P<0.05). * Significant difference between two groups. All data demonstrated as mean±SD. Data considered significant at the level of p<0.05.

## 4. Discussion

To our knowledge, this study is the first randomized double blind clinical trial for evaluation of omega-3 as a supplemental therapy with conventional antipsychotic drugs among Iranian schizophrenic patients. Both omega-3 supplement therapy and standard conventional drug therapy were effective in reduction of PANSS score during 8 week treatment in comparison to baseline scores (P<0.05). We also found that treatment with omega-3 supplement in addition to standard treatment for schizophrenia, significantly decreased PANSS total score after week 6 and general score after week 4 of treatment (p<0.05). Although this difference was significant until the end of study for total and general scores, no significant difference was found between two groups for positive and negative scores (P>0.05).

Recent studies found that levels of omega-3 and omega-6 are decreased in the brains of schizophrenic patients. They also found that levels of these fatty acids are low in post-mortem brain, red blood cells and brains of patients with schizophrenia.(Horrobin, 1991; Peet, Laugharne, Horrobin, & Reynolds, 1994; Yao, Leonard & Reddy, 2000)

In agreement to our findings, there are some studies examining a therapeutic role of omega-3 supplementation in patients with schizophrenia. Wolkin et al. in 1986 evaluated efficacy of linolenic acid vs. placebo as supplementation therapy in 6 week treatment of tardive dyskinesia among schizophrenic patients. They found no statistical difference between two groups and no antidyskinetic efficacy by c-linolenic acid was found (Wolkin et al., 1986)

During a 16 weeks placebo-controlled trial of ethyl EPA (eicosapentaenoic acid) supplementation for residual symptoms and cognitive impairment in schizophrenia, fenton et al. found that EPA supplement therapy can improve positive and negative symptoms as well as in patients with schizophrenia. Otherwise, no statistical differences were found in positive and negative symptoms between groups (Fenton, 2001).

Two double blind placebo controlled pilot studies of EPA in the treatment of schizophrenia conducted by Peet et al. in 2001. In their 3 months long study, they have tested the effects of EPA vs. Docosahexaenoic acid (DHA) vs. placebo (which is an omega-3 fatty acid that is a primary structural component of the human brain cerebral cortex, sperm, testicles and retina) on schizophrenic symptoms. They found that both EPA and DHA supplement therapy reduced PANSS score while EPA made a greater reduction in positive symptoms in comparison to DHA (3).

Seemingly, Emsley et al. in 2002 found that 12 weeks supplement therapy with omega-3 among schizopherenic patients leads to a significantly greater decrease in positive and negative symptoms in comparison to conventional antipsychotics (Emsley et al., 2002)

In contrast to our findings, Emsly et al. 2006 demonstrated that 12 weeks treatment of schizophrenia with omega-3 supplement cannot significantly decrease tardive dyskinesia. However, our results emphasizes on significant efficacy of omega-3 in treatment of general psychopathologic symptoms of schizophrenia.

Adverse effects from the addition of omega-3 fatty acids in to conventional antipsychotic therapy were negligible except for gastrointestinal symptoms like fishy eructation, nausea, and loose stools. These outcomes suggest that the use of omega-3 fatty acids is safe and do not interfere with the effectiveness or enhancement of adverse events among antipsychotics (Morrison et al., 2004; Peet & Horrobin, 2002). On the other hand, metabolic changes, sexual dysfunction and weight gain associated with the use of antipsychotics, which are often not acceptable for young patients, made omega-3 fatty acid as a beneficial supplement in treatment of schizophrenia in order to help the conventional antipsychotics acts well or even help psychiatrists to reduce the daily dose of these drugs.

## 5. Conclusion

We found that using omega-3 fatty acid, as a supplement therapy, could be helpful in treatment of symptoms among schizophrenic patients. Considering the wide range of side effects of conventional antipsychotics, omega-3 could be a potential supplement for reducing the total dose of these drugs, which could help the patients have a better life.
